# Morphometry of the wings of *Anopheles aquasalis* in simulated scenarios of climate change

**DOI:** 10.1590/0037-8682-0454-2023

**Published:** 2024-04-05

**Authors:** Wilsandrei Cella, Rubens Celso Andrade da Silva, Paulo Filemon Paolucci Pimenta, Wuelton Marcelo Monteiro

**Affiliations:** 1 Universidade do Estado do Amazonas, Centro de Estudos Superiores de Tefé, Tefé, AM, Brasil.; 2 Universidade Paranaense, Programa de Pós-graduação Stricto Sensu em Ciência Animal com Ênfase em Produtos Bioativos, Umuarama, PR, Brasil.; 3 Fundação de Medicina Tropical Dr. Heitor Vieira Dourado, Manaus, AM, Brasil.; 4 Fundação Oswaldo Cruz, Instituto de Pesquisas René Rachou, Belo Horizonte, MG, Brasil.; 5 Universidade do Estado do Amazonas, Programa de Pós-Graduação Stricto Sensu em Medicina Tropical, Manaus, AM, Brasil.

**Keywords:** Anopheles, Climate change, Malaria, Infectious diseases

## Abstract

**Background:**

Climate change has significant implications on ecosystems. We verified the effects of climate change on the malaria vector *Anopheles aquasalis* using simulated climate change scenarios (SSCCs).

**Methods:**

An experimental model was designed for SSCCs, which composed of air-conditioned 25 m^3^ rooms.

**Results:**

The wing size was significantly different between SSCCs. A colony of *Anopheles aquasalis* could not be established in extreme scenarios.

**Conclusions:**

Increases in temperature and CO_2_ in the atmosphere may modify the global epidemiology of malaria, marking its emergence in currently malaria-free areas.

The severity of climate change and its effects on different sectors of human activity are controversial subjects. However, the significant impacts of climate change on public health seem inevitable, especially in the appearance and spread of new diseases, with an emphasis on vector-borne diseases (VBDs)^1^ ^,^ ^2^.

Arthropods exhibit extraordinary biological diversity and are found worldwide in all environments. They are of great ecological and economic importance, especially in food production^1^. However, mosquitoes belonging to the order Diptera (approximately 3,600 species) can transmit a multitude of diseases^2^. Major disease pathogens that are spread worldwide by mosquito vectors to the human population include arboviruses (Zika, dengue, chikungunya, and yellow fever viruses)^3^ and *Plasmodium* spp*.*, which are the etiological agents of human malaria. Among VBDs, malaria is one of the main causes of global human mortality^4^. These mosquito-transmitted diseases have a close epidemiological relationship with climate change^5^. 

In recent decades, studies have demonstrated the effects of climate change on many species, including changes in their geographic distribution, seasonal activity, migration patterns, abundance and intraspecific interactions^6^. This phenomenon has caused severe environmental imbalances and, consequently, the resurgence of existing diseases and/or the emergence of new diseases^7^.

Currently, the biggest challenge is predicting the impacts of climate change on vector species and how this phenomenon will affect tropical diseases, including their spread to Old World countries^5^. Hence, establishing an experimental model is imperative for future studies on the impact of climate change on mosquito vectors of severe human diseases.

Morphological characteristics are important for demonstrating the adaptations developed as evolutionary strategies for this species. In insects, the wing is a highly relevant structure and allows the identification of several ecological aspects inherent to the species^8^. It is a structure that is widely used for taxonomic identification. However, in the present study, the wing was used to correlate the body sizes of the anopheline species. According to Vaz, Tavares, and Lomônaco^9^, insect size can be estimated by correlating it with wing size.

This study aimed to verify the differences in the dimensions (length and width) of *An. aquasalis* wings under the simulated scenarios of climate change (SSCCs) to predict the effects of climate change on the size of malarial vector insects. This species is an important malaria vector in the Americas. It is an easy-to-handle species in the laboratory, colonized in insectariums many years ago, and has been used as an experimental model to study the interaction of malaria vectors with *Plasmodium* species.

This study was conducted at the Laboratory of Ecophysiology and Molecular Evolution of the Amazonian Aquatic Biota Adaptation Studies Center (ADAPTA) of the National Institute for Amazonian Research (INPA) in Manaus, Amazonas, Brazil.

The SSCCs were replicated in three of the four 25 m^3^ air-conditioned rooms (microcosms), which were independently controlled by a computer. Every two minutes, CO_2_, temperature, and relative humidity (RH) were recorded using sensors installed in a tower in a natural forest located close to the municipality of Manaus, Amazonas, Brazil. The variables were reproduced in a control room in real-time. For the other three microcosms reproduced the predictions of IPCC^11^ based on the control room were reproduced. The photoperiod in the rooms was set to 12/12 h. The environmental variables in the experimental rooms were obtained using Data Loggers Novus^®^ equipment. The Fieldlogger Software 1.5.2 Novus^®^ was used for data management, and the data were processed and analyzed in computerized spreadsheets using the Microsoft Excel 2016^®^ program.

The study period was from July to November 2020, a season considered the “Amazon summer,” with high temperatures (≅ 27.70 °C). According to the IPCC estimates of air temperature and CO_2_ concentration for the year 2100^11^, the rooms were named: i) Mild - B1: increases of ≅ 1.5 °C and ≅ 220 ppm CO_2_ in relation to the control condition; ii) Moderate - A1B: increases of ≅ 3.0 °C and ≅ 420 ppm of CO_2_ in relation to the control condition; and iii) Extreme - A2: increase of ≅ 4.5 °C and ≅ 870 ppm of CO_2_ in relation to the control condition. All SSCCs mirrored the environmental conditions of the control room, which had real-time environmental conditions of ≅ 27.70 °C and a CO_2_ concentration of ≅ 398.81 ppm.

Approximately 2,500 eggs of well-colonized *An. aquasalis* were obtained from the Insectary of the Doctor Heitor Vieira Dourado Tropical Medicine Foundation (FMT-HVD), Manaus, Amazonas, Brazil. The eggs were evenly divided and placed in plastic trays (20.5 x 30.5 x 6.0 cm) containing 600 mL of water and 12 mL of saline solution (10%). Three trays containing 150 larvae each were placed in all the rooms. All the larvae were fed daily with commercial fish feed (Tetramin Gold^®^), sieved with granulometric sieves of 125, 125, 300, and 300 µm for the stages L1, L2, L3, and L4, respectively (Supplementary Table 1). Adult mosquitoes were maintained in a 10% sucrose solution, provided *ad libitum* ^10^ *.*

Three to five-day-old *An. aquasalis* females were maintained under sucrose restriction for 24 h before a blood meal. For blood feeding, female mosquitoes were allowed to feed directly on the skin of Balb/c mice (*Mus musculus*) for 45 min inside each room in the dark. Fully engorged mosquitoes were separated and maintained in sucrose solution supplied *ad libitum* until they had thoroughly digested the bloodmeal. They were then immediately placed in a container designed for egg laying. The eggs were evenly divided and placed in plastic trays as described above. This operation was repeated until four generations (G4) of *An. aquasalis* were obtained.

The project was submitted to the Committee on Ethics on the Use of Animals (CEUA) at INPA and approved under opinion no. 015/2020, SEI 01280.000226/2020-26.

Females in G4 of *An. aquasalis* from each room were randomly separated and used for wing measurements. The mosquitoes were euthanized by freezing at −20 °C for 40 min. The right wing of each insect was excised using an entomological stylet. The length and width were measured according to Vaz, Tavaves, and Lomônaco^9^. A stereomicroscope (Zeiss, Stemi 508^®^) coupled to a camera (AxionCam 105 color^®^) and Zeiss blue version^®^ software were used (Figure 1). The measurements were performed in triplicates.

**FIGURE 1:**
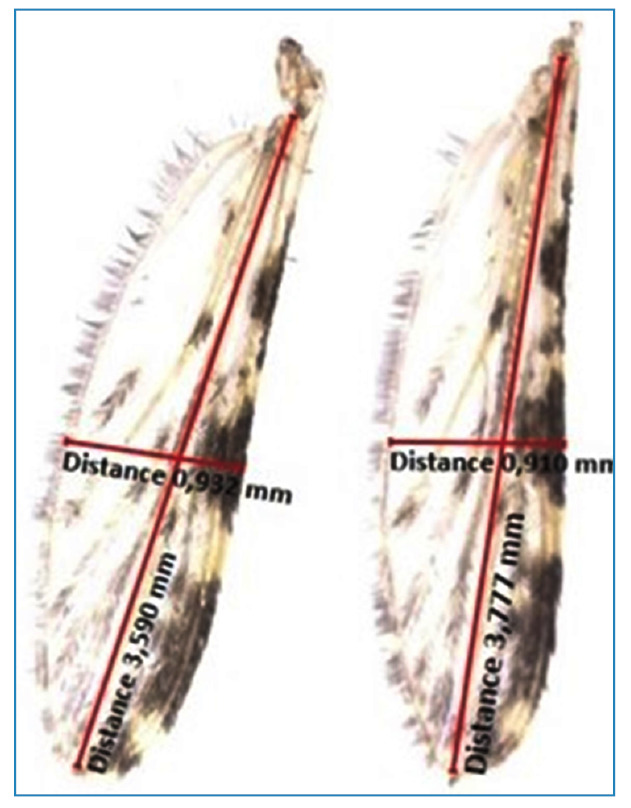
Points used to estimate the length and width of right wing of *An. aquasalis.*

The results provide robust evidence regarding the differences in the wing sizes of *An. aquasalis* under the simulated scenarios. In scenario A1B, wing length was shorter than those in the other scenarios and presented significant differences (Supplementary Figure 1A). However, when the width measurements were analyzed, no significant differences were observed between the simulated scenarios and the control (Supplementary Figure 1B). The relationship between the length and width measurements showed significant differences between scenarios B1 and A1B (Supplementary Figure 1C and Table 1).

**TABLE 1:** Analysis of variance for wing morphometry of female *An. aquasalis* in the different simulated scenarios of climate change (SSCCs).

Millimeter (mm)	Scenarios		
	Control	Mild - B1	Moderate - A1B
Length	3.6817 ± 0.1069 ^A^	3.6978 ± 0.1381 ^A^	3.5608 ± 0.1298 ^B^
Width	0.8898 ± 0.0540 ^A^	0.9192 ± 0.0478 ^A^	0.9019 ± 0.0867 ^A^
Length × Width	3.277 ± 0.238 ^AB^	3.400 ± 0.231 ^A^	3.216 ± 0.368 ^B^

Different letters on the same line indicate a significant difference in wing size between SSCCs, according to Tukey’s test (p < 0.05). The extreme microcosm A2 is not included in the table as colonization was not carried out owing to the death of all the *An. aquasalis* in F0.

In scenario A2, which had extreme abiotic variables, the insects did not survive after the pupal phase, and 100% mortality occurred in the generation F0. After three attempts in triplicate, we could not colonize *An. aquasalis* under these simulated conditions (Table 2). All simulated scenarios presented significant differences in the abiotic variables of temperature, CO_2_ concentration, and relative humidity with p < 0.000 (Table 2).

**TABLE 2:** Mean values of abiotic variables in simulated scenarios of climate change (SSCC).

Abiotic variables	Control	Mild	Moderate	Extreme	p
		B1	A1B	A2	
**CO**_2_ **(ppm)**					
Mean	398.81 ± 25.44 ^D^	604.61 ± 63.12 ^C^	836.92 ± 89.10 ^B^	1,298.78 ± 97.53 ^A^	< 0.000
Min. - Max.	273.00-620.00	481.00-1355.00	579.00-1,309.00	839.00-2,049.00	
Confidence interval	(397.86-399.76)	(603.65-605.55)	(835.90-837.87)	(1,299.79-1,301.85)	
**Temperature (ºC)**					
Mean	27.70 ± 1.95 ^D^	28.94 ± 2.02 ^C^	29.48 ± 1.93 ^B^	32.00 ± 2.15 ^A^	< 0.000
Min. - Max.	23.90-33.50	23.70-36.50	24.20-37.10	26.40-39.60	
Confidence interval	(27.67-27.72)	(28.91-28.96)	(29.44-29.50)	(31.97-32.03)	
**RH%**					
Mean	76.54 ± 6.71 ^B^	70.81 ± 6.36 ^D^	77.04 ± 7.15 ^A^	75.56 ± 5.47 ^C^	< 0.000
Min. - Max.	45.10-90.10	46.70-85.80	51.80-90.50	48.20-86.90	
Confidence interval	(76.45-76.62)	(70.72-70.89)	(76.95-77.12)	(75.32-75.50)	

Different letters on the same line indicate significant differences between the abiotic variables of different SSCCs via Tukey’s test (p < 0.05). Confidence interval (𝛼 = 0.05); colonization in the extreme microcosm A2 was not carried out as all the *An. aquasalis* died in F0.

This experimental study reports the first successful introduction, colonization, and maintenance of *An. aquasalis* for four consecutive generations in two SSCCs (Mild - B1 and Moderate - A1B), as foreseen in the fourth report of the Intergovernmental Panel on Climate Change for the year 2100^11^.

Notably, in the extreme microcosm (A2), three trials were conducted in triplicate, and *An. Aquasalis* could not be colonized because all insects of the F0 generation died before the tenth day after the pupation phase, thus impeding the experiment in this environment. These findings corroborate those of an experimental study by Murdock, Sternberg, and Thomas^12^, who found a considerable increase in adult mortality in *An. stephensi* and *An. gambiae* at temperatures above 30 °C. Similar studies have confirmed that the mortality rate and survival time of anophelines are proportional to increases in temperature during the juvenile and adult phases^13^.

The wings were chosen for measurements because they are flat structures and easy to handle, thus allowing for greater precision in obtaining data. Normally, wing morphometric analyses are used as tools for taxonomic identification of mosquitoes, and several methodologies have been used by different authors for this purpose^8^. However, to our knowledge, this is the first study to use *Anopheles* wings to predict the effects of climate change based on the size of malaria-carrying insects.

The values obtained from the measurements of *An. aquasalis* wings showed differences among the three SSCCs (Table 1), indicating that the insects were susceptible to the abiotic variables (CO_2_, temperature, and RH) in the different microcosms. Beck-Johnson et al.^14^ asserted that mosquitoes are very sensitive to climatic conditions that directly interfere with their development. When the widths of the mosquito wings from the three SSCCs were evaluated, as along with the lengths of the wings of the insects from the control and mild microcosms (B1), no differences were observed (Supplementary Figure 1A and Supplementary Figure 1B). However, there were significant differences between the abiotic variables of the SSCCs (Table 2).

Notably, insects colonized in the moderate microcosm (A1B) had shorter wings than those in the control (Supplementary Figure 1A). When analyzing the relationship between length and width, the mosquitoes in scenarios B1 and A1B also showed significant differences (Supplementary Figure 1C), demonstrating that *An. aquasalis* are sensitive to climate change. 

According to Di Mare and Corseuil^15^, long-distance displacements requires greater muscle mass. Thus, a correlation between wing size and insect has been estimated^9^. Therefore, the smaller the wing of the insect, the smaller its body structure, and consequently, the smaller its weight, and the shorter the distance it can travel, thus limiting flight to short distances. Gene expression may change despite the unique genotype of each living organism, resulting from phenotypic interactions affected by environmental conditions. Thus, climate change can definitively alter the epidemiology of malaria in smaller and strictly peculiar geographic regions, especially in hot places such as the Amazon, owing to the probable consequences of climate change in the *Anopheles* phenotype.

Therefore, climate change predicted for the year 2100^11^ could definitively change the global epidemiology of malaria, with an increase in cases in colder regions that are currently considered free of the disease according to some of the predictions^5^ ^,^ ^7^ ^,^ ^12^ ^,^ ^13^. Our results reinforce this perspective of change in malaria epidemiology, highlighting significant differences in the size of insect wings between SSCCs, as well as the impossibility of colonizing extreme scenarios.

This study had some limitations. Our approach was based on traditional morphometry, which uses linear distance measurements between anatomically homologous points. As such, we recommend that future research on SSCCs consider the use of geometric morphometry using specialized software. In addition, we suggest parallel molecular studies to clarify the gene expression related to vector susceptibility under different climatic conditions.

In conclusion, our results showed significant differences in the size of *An. aquasalis* wings when reared in the mild (B1) and moderate (AB1) scenarios and in the control. In the extreme scenario (A2), 100% of the F0 generation died after the pupation phase, making it impossible to establish *An. aquasalis* colonies in this microcosm. Therefore, we conclude that temperature is a limiting factor for the survival of this species and that an increase in temperature and CO_2_ concentration in the atmosphere, as predicted to happen by the end of this century, could significantly modify the global epidemiology of malaria. However, further experimental studies are needed to better understand the behavior of the vector and etiological agent of malaria in simulated climate change scenarios.

## References

[1] Intergovernmental Panel on Climate Change (IPCC) (2023). Climate Change 2023: Synthesis Report.

[2] Cáceres SB (2012). Climate change and animal diseases: making the case for adaptation. Anim Health Res Rev.

[3] McGregor BL, Connelly CR (2021). A review of the control of Aedes aegypti (Diptera: Culicidae) in the Continental United States. J Med Entomol.

[4] Biddau M, Sheiner L (2019). Targeting the apicoplast in malaria. Biochem Soc Trans.

[5] Caminade C, McIntyre KM, Jones AE (2019). Impact of recent and future climate change on vector-borne diseases. Ann N Y Acad Sci.

[6] Intergovernmental Panel on Climate Change (IPCC) (2014). Climate Change 2014: Synthesis Report.

[7] El-Sayed A, Kamel M (2020). Climatic changes and their role in emergence and re-emergence of diseases. Environ Sci Pollut Res Int.

[8] Lorenz C, Almeida F, Almeida-Lopes F, Louise C, Pereira SN, Petersen V (2017). Geometric morphometrics in mosquitoes: What has been measured?. Infect Genet Evol.

[9] Vaz LAL, Tavares MT, Lomônaco C (2004). Diversidade e tamanho de himenópteros parasitóides de Brevicoryne brassicae L. e Aphis nerii Boyer de Fonscolombe (Hemiptera: Aphididae). Neotrop Entomol.

[10] Villarreal-Treviño C, Vásquez GM, López-Sifuentes VM, Escobedo-Vargas K, Huayanay-Repetto A, Linton YM (2015). Establishment of a free-mating, long-standing and highly productive laboratory colony of Anopheles darlingi from the Peruvian Amazon. Malar J.

[11] Intergovernmental Panel on Climate Change (IPCC) (2007). Climate Change 2007: Synthesis Report.

[12] Murdock CC, Sternberg ED, Thomas MB (2016). Malaria transmission potential could be reduced with current and future climate change. Sci Rep.

[13] Agyekum TP, Arko-Mensah J, Botwe PK, Hogarh JN, Issah I, Dwomoh D (2022). Effects of Elevated Temperatures on the Growth and Development of Adult Anopheles gambiae (s.l.) (Diptera: Culicidae) Mosquitoes. J Med Entomol.

[14] Beck-Johnson LM, Nelson WA, Paaijmans KP, Read AF, Thomas MB, Bjørnstad ON (2013). The effect of temperature on Anopheles mosquito population dynamics and the potential for malaria transmission. PLoS One.

[15] Di Mare RA, Corseuil E (2004). Morfometria de Papilioninae (Lepidoptera, Papilionidae) ocorrentes em quatro localidades do Rio Grande do Sul, Brasil. II. Relação entre partes do corpo, aerodinâmica de vôo e tipos de asas. Rev Bras Zool.

