# Supplementary material for: Morphometry of the wings of Anopheles aquasalis in simulated scenarios of climate change
Source: Rev Soc Bras Med Trop. 2024 Apr 5;57:e00704-2024. doi: 10.1590/0037-8682-0454-2023 (PMC11000507; doi:10.1590/0037-8682-0454-2023)
Supplement: Supplementary file 1 [file 1678-9849-rsbmt-57-e00704-2024-supp1.pdf]

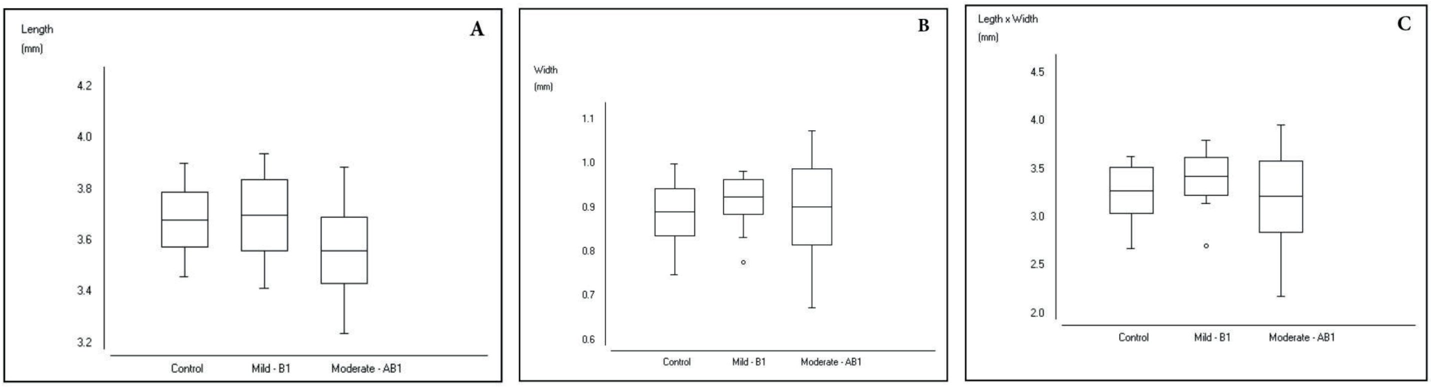

SUPPLEMENTARY FIGURE 1: Wing sizes of *An. aquasalis* in simulated scenarios of climate change. **(A)** Length measurements (mm). **(B)** Width measurements (mm). **(C)** Length × width measurements (mm).
