# Supplementary material for: Morphometry of the wings of Anopheles aquasalis in simulated scenarios of climate change
Source: Rev Soc Bras Med Trop. 2024 Apr 5;57:e00704-2024. doi: 10.1590/0037-8682-0454-2023 (PMC11000507; doi:10.1590/0037-8682-0454-2023)
Supplement: Supplementary file 2 [file 1678-9849-rsbmt-57-e00704-2024-supp2.pdf]

SUPPLEMENTARY TABLE 1: Daily water volume and feed amount per plastic tray, when supplied *ad libitum*.

Larvae	(n)	Water/mL	Plastic tray (cm)	Fish feed/Gram
Stages L_1-L_2	150	600	30.5 × 20.5 × 6.0	0.0423
Stages L_3-L_4	150	600	30.5 × 20.5 × 6.0	0.127
